# Quantifying the effects of anomalies of temperature, precipitation, and surface water storage on diarrhea risk in Taiwan

**DOI:** 10.4178/epih.e2023024

**Published:** 2023-02-15

**Authors:** Gerry Andhikaputra, Ayushi Sharma, Amir Sapkota, Hao He, Yu-Kai Lin, Li-Wen Deng, Yu-Chun Wang

**Affiliations:** 1 Department of Environmental Engineering, Chung Yuan Christian University College of Engineering, Zhongli, Taiwan; 2 Department of Epidemiology and Biostatistics, University of Maryland School of Public Health, College Park, MD, USA; 3 Department of Atmospheric and Oceanic Science, University of Maryland, College Park, MD, USA; 4 Department of Health and Welfare, University of Taipei College of City Management, Taipei, Taiwan; 5 Research Center for Environmental Changes, Academia Sinica, Taipei, Taiwan

**Keywords:** Diarrhea, Weather anomaly, Extreme weather, Climate change

## Abstract

**OBJECTIVES:**

Diarrheal disease continues to be a significant cause of morbidity and mortality. We investigated how anomalies in monthly average temperature, precipitation, and surface water storage (SWS) impacted bacterial, and viral diarrhea morbidity in Taiwan between 2004 and 2015.

**METHODS:**

A multivariate analysis using negative binomial generalized estimating equations was employed to quantify age-specific and cause-specific cases of diarrhea associated with anomalies in temperature, precipitation, and SWS.

**RESULTS:**

Temperature anomalies were associated with an elevated rate of all-cause infectious diarrhea at a lag of 2 months, with the highest risk observed in the under-5 age group (incidence rate ratio [IRR], 1.03, 95% confidence interval [CI], 1.01 to 1.07). Anomalies in SWS were associated with increased viral diarrhea rates, with the highest risk observed in the under-5 age group at a 2-month lag (IRR, 1.27; 95% CI, 1.14 to 1.42) and a lesser effect at a 1-month lag (IRR, 1.18; 95% CI, 1.06 to 1.31). Furthermore, cause-specific diarrheal diseases were significantly affected by extreme weather events in Taiwan. Both extremely cold and hot conditions were associated with an increased risk of all-cause infectious diarrhea regardless of age, with IRRs ranging from 1.03 (95% CI, 1.02 to 1.12) to 1.18 (95% CI, 1.16 to 1.40).

**CONCLUSIONS:**

The risk of all-cause infectious diarrhea was significantly associated with average temperature anomalies in the population aged under 5 years. Viral diarrhea was significantly associated with anomalies in SWS. Therefore, we recommend strategic planning and early warning systems as major solutions to improve resilience against climate change.

## GRAPHICAL ABSTRACT

[Fig f4-epih-45-e2023024]

## INTRODUCTION

Diarrheal diseases represent the second-leading cause of mortality among children in Africa and South-East Asia, accounting for 25% of under-5 mortality [1]. Despite significant improvements in water, sanitation, and hygiene and rotavirus vaccination, diarrhea-specific mortality continues to be a persistent issue [2], accounting for 9% (0.478 million) of pediatric death globally [3]. The etiological agents of pediatric diarrhea include bacteria, viruses, and parasites [4], with a recent study from China showing bacterial pathogens as the predominant agent (32.3%) [5]. Globally, the most common bacteria associated with diarrheal diseases include *Escherichia coli*, followed by *Shigella*, *Salmonella*, *Campylobacter* (primarily associated with childhood diarrhea), *Yersinia*, and *Clostridium* spp. [6].

Since warmer temperatures can promote bacterial growth and increases in precipitation can enhance the fecal-oral route of exposure, prior studies have suggested that ongoing climate variability and change may worsen the diarrheal disease burden globally [7-9]. Prior studies have revealed that extreme temperatures, excessive rainfall, and drought increase the risk of infectious diseases, with significant heterogeneity observed among different geographic regions [10-12]. A recent study from Nepal found that the burden of under-5 diarrheal disease in Kathmandu was positively associated with warmer temperatures, with the monthly number of diarrheal cases increasing by 8.1% per 1°C increase in maximum temperature [13], with a considerably higher risk observed during the monsoon season and La Niña periods [14]. Likewise, a study from Taiwan reported that the incidence of diarrhea was associated with warming temperatures [15].

Previous studies also reported that drought can escalate the risk of infectious diseases [12,16,17]. A study conducted in sub-Saharan Africa observed an increased incidence of cholera during drought periods [16], while others have linked longer droughts with increased cholera risks [16,18]. Interestingly, heavy rainfall events are also reported to be a risk factor for diarrheal disease [10], with considerably higher risk of diarrhea when a dry period is followed by heavy rainfall [11]. Others have shown floods or heavy rainfall are more strongly associated with cholera outbreaks [16] and extreme bacillary dysentery [19]. This highlights the importance of flooding in the spread of infectious diseases. However, even within small geographic areas, flooding can be highly heterogeneous based on hydrological runoff and specific elevation. The recently developed Global Flood Monitoring System (GFMS) provides estimates of surface water storage (SWS) [20] that provide an indirect assessment of high-resolution flooding data that may be useful in epidemiological investigations of flooding events and the infectious disease burden. SWS is an estimate of surface water depth (mm) above the land, reflecting recent flood occurrences and their intensities. It includes all surface water constrained in water bodies and overflowing to surrounding floodplains [21]. However, no studies to date have evaluated whether SWS is associated with the diarrheal disease burden.

While number studies have linked weather phenomena (daily temperature, precipitation, and flooding) with burden of diarrheal disease, there is a paucity of data regarding how long-term changes in such weather phenomena impact the disease burden. To address these shortcomings, increasingly many epidemiological studies have begun to investigate this question using the frequency of extreme weather events and weather anomalies as exposure metrics, which are more relevant in the context of climate change [9,22,23]. In this study, we investigated how long-term anomalies in temperatures, precipitation, and SWS affected cause-specific diarrhea in all ages and the under-5 population in Taiwan using 12 years (2004-2015) of surveillance data.

## MATERIALS AND METHODS

### Study area

Taiwan, a subtropical island (150 km× 350 km) with 23 million people [24,25], is located in one of the main paths of tropical cyclones in the western North Pacific Ocean and has been experiencing drastic impacts of climate change. The southern part of Taiwan has experienced increases in minimum temperature at the rate of 2.98°C per 100 years [26,27]. A recent study from Taiwan reported that more than 4,500 disability-adjusted life years (DALYs) were attributable to foodborne illnesses resulting from non-typhoid *Salmonella*, norovirus, and *Vibrio parahaemolyticus* [28].

### Data sources

**Figure 1.**
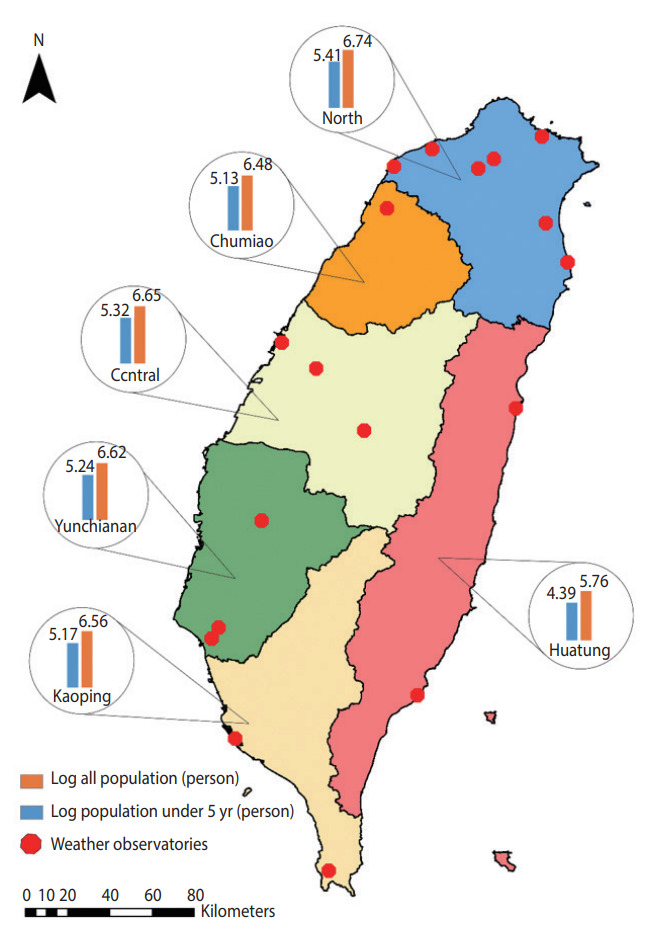
Distribution of weather observatories and age-specific log population across six regions in Taiwan.

We obtained the monthly number of emergency room and outpatient visit records (2004-2015) of cause-specific diarrheal disease cases from the National Health Insurance (NHI) database of the Ministry of Health and Welfare for the 6 regions of Taiwan (North, Chumiao, Central, Yunchianan, Kaoping, and Huatung) (Figure 1). The NHI provides equal-access health care in Taiwan and covers more than 99% of Taiwan’s population [29]. The overall identification numbers were replaced by surrogate numbers to protect patients’ privacy. This study used the ninth and 10th revisions of the International Classification of Diseases (ICD-9 and ICD-10) codes to identify diarrheal disease cases. These included bacterial cases (*V. cholera*, *Salmonella* spp., *E. coli*, *Campylobacter enteritis*, *Yersinia enterocolitica*, *Clostridium difficile*, and other bacteria [ICD-9: 001, 003, 008 and ICD-10: A00, A02, A04]), viral cases (rotavirus, adenovirus, and Norwalk virus [ICD-9: 8.61-8.63 and ICD-10: A08.0, A08.2, A08.1]), and all other infectious diarrheal cases (ICD-9: 001-009 and ICD-10: A00-A09).

We obtained weather data from the Taiwan Central Weather Bureau, including average temperature (°C) and precipitation (mm) for 18 weather stations located in the 6 regions of Taiwan for the same period (Figure 1). Weather data were aggregated from hourly resolution to monthly resolution for the analysis to match the temporal resolution of the outcome measures. Likewise, we extracted SWS data from the Global Flood Monitoring System (GFMS), which is freely available from the University of Maryland (http://flood.umd.edu/). The population data for each location stratified by age group were retrieved from National Statistics, Republic of China, which provides open access to the yearly population. Further detailed information about population data is available on its official portal (https://eng.stat.gov.tw/).

### Anomalies in weather variables

To evaluate the effect of changing climate on infectious diarrheal disease, we adopted anomalies in weather variables as the exposure metric, since they reflect changes in the historical context (climate) rather than direct measurements of weather variables. More specifically, we decided to focus on long-term monthly anomalies instead of weekly or daily variability. We aggregated weather data and health outcomes onto a monthly temporal scale, since we believe that a monthly scale would show distinct patterns of anomalies compared to a daily or weekly scale. To achieve this, we first calculated a 30-year baseline (1980-2010) to obtain a longterm monthly average for each calendar month specific to each of the 6 regions. We then calculated the anomalies for our study period (2004-2015) by subtracting the monthly mean weather data from their respective long-term averages.

### Statistical analysis

We used a multivariate generalized estimating equation (GEE) model with negative binomial regression [30] to examine the association between anomalies of +1°C temperature, +1 mm precipitation, and +1 mm SWS and monthly cause-specific diarrheal disease cases. The following model was considered:

Log[Y]~(temperature,lag)+(precipitation,lag)+(SWS,lag)+(season)+(time)+Lunar New Year event+offset(population)

The study also included the effect of seasonality and Lunar New Year events in the model. Population statistics were included in the model as an offset variable. Based on the hydrological cycle, the seasons in Taiwan are classified into 5: winter (December-January), spring (March-April), *mei-yu* (East Asian rainy season; May-June), typhoon (July-August), and autumn (September-November) [31]. We created a binary variable to indicate Lunar New Year events by labeling months with a Lunar New Year event as “1” and put it as a predictor in the model. We included lag structures of up to 2 months (0-2 months) to capture the delayed effect of the predictors on the disease rates. The risks in statistical analyses were reported as incidence rate ratios (IRRs) with 95% confidence intervals (CIs) and interpreted as showing the risk for every 1-unit increase in the weather anomaly variables. The IRR has been widely used in epidemiology to report whether the exposure to dependent variables can increase or decrease the risk of the incidence of various conditions [14,32].

We also categorized the predictor variables into 5 groups based on their percentile distribution and used the normal category as the reference group for the analysis. For example, average temperature anomalies were categorized as extremely cold (< 5th), cold (≥ 5-< 30th), normal (≥ 30-≤ 70th), hot (> 70-≤ 95th), and extremely hot temperature (> 95th). The categorization for precipitation and SWS followed suit. The detailed classification is presented in Supplementary Material 1. We used exchangeable correlations and clustered the data based on the 6 regions in Taiwan. We tested several model combinations, which included univariate, multivariate, and model with interaction effects, and selected a model based on the lowest quasi-information criterion (Supplementary Material 2).

### Ethics statement

The study was approved by Institutional Review Board at the Chung Yuan Christian University and University of Maryland. All methods were carried out in accordance with relevant guidelines and all protocols were approved by Taiwan National Health Research Institutes (code: EC1090703-F-E).

## RESULTS

### Descriptive statistics

**Figure 2.**
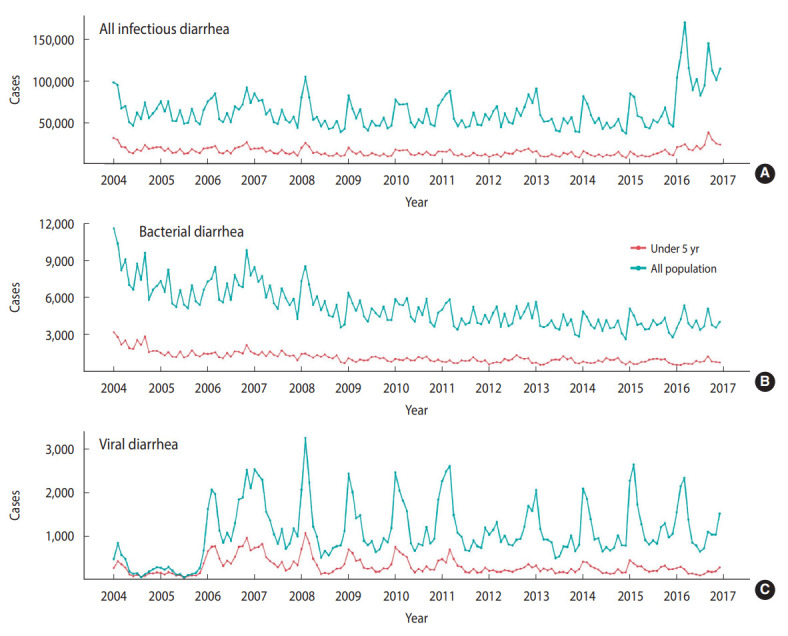
Age-specific trends of monthly all-cause infectious (A), bacterial (B), and viral diarrhea (C) cases from 2004 to 2015 in Taiwan.

A total of over 10 million diarrheal disease cases were reported from 2004 to 2015 in Taiwan (Table 1). The monthly average incidence rate per 100,000 population was 254 for all infectious diarrhea, 25 for bacterial diarrhea, and 5 for viral diarrhea. The monthly trends of cause-specific diarrhea cases by age from 2004 to 2015 are illustrated in Figure 2. An upward trend was observed for allcause infectious diarrhea cases, while bacterial and viral diarrhea cases showed downward trends.

**Figure 3.**
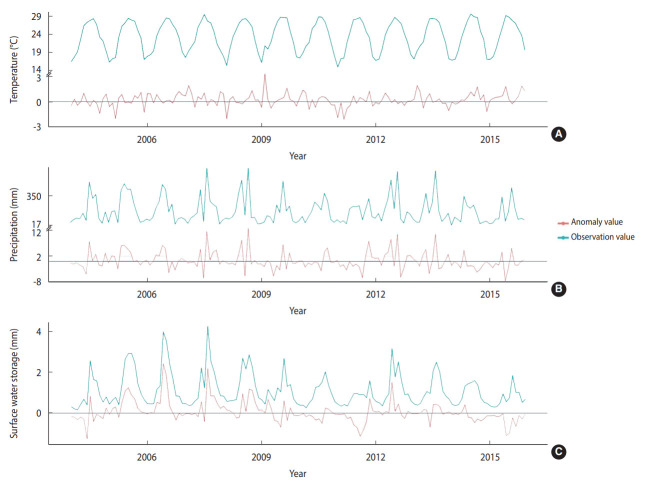
Monthly observations and anomalies of temperature (A), precipitation (B), and surface water storage (C) from 2004 to 2015 in Taiwan.

The monthly average temperature during the study period was 23.24°C across the 6 regions of Taiwan. The monthly mean values for precipitation and SWS were 178.24 mm and 1.11 mm, respectively. The temperature ranged from 14.8°C to 20.7°C during the cold months (December to February) and 26.5°C to 29.5°C during the hot months (June to August) (Figure 3). The precipitation and SWS showed similar trends with the highest values of 662.7 mm and 4.2 mm, respectively, from June to September. The mean monthly average temperature, precipitation, and SWS, as well as their respective anomalies, are presented in Table 1, while their temporal trends are depicted in Figure 3.

### Association between cause-specific diarrhea and weather anomalies

The results from the univariate analysis of the associations between anomalies in temperature, precipitation, and SWS and the risk of diarrheal disease are depicted in Supplementary Material 3. The associations between diarrheal disease rates and 1-unit increases in temperature precipitation, and SWS anomalies in Taiwan are presented in Table 2. A +1°C anomaly in average temperature (2-month lag) was associated with an increased risk of infectious diarrhea among all age groups (IRR, 1.03; 95% CI, 1.01 to 1.05) as well as in the under-5 age group (IRR, 1.03; 95% CI, 1.01 to 1.07). A similar association was observed for bacterial diarrhea in the under-5 age group at lags of 1 month and 2 months (IRR, 1.04; 95% CI, 1.01 to 1.07 for both), but not for viral diarrhea. However, no association was found between a +1 mm anomaly in precipitation and infectious diarrhea in Taiwan. Interestingly, a +1 mm anomaly in SWS was consistently associated with an increased risk of viral diarrhea, irrespective of the lag structure. For example, increases in viral diarrhea in the under-5 age group ranged from 12% (IRR, 1.12; 95% CI, 1.00 to 1.25) at lag 0 to 27% at a lag of 2 months (IRR, 1.27; 95% CI, 1.14 to 1.42), while the corresponding values among the entire population ranged from 15% (IRR, 1.15; 95% CI, 1.03 to 1.28) to 22% (IRR, 1.22; 95% CI, 1.09 to 1.36) at a lag of 2 months. This study did not find any significant effects between anomalies in SWS and all-cause infectious diarrhea or bacterial diarrhea. We also tested for interactions between SWS and temperature/precipitation anomalies and observed evidence of a limited interaction (Supplementary Material 4). However, we found positive interactions in the association between temperature and SWS on the risk of all-cause infectious diarrhea among all age groups at lag 0 (IRR, 1.08; 95% CI, 1.03 to 1.14). Detailed results can be seen in Supplementary Material 4.

### Association between cause-specific diarrhea and extreme weather events

Table 3 shows the associations between exposure to various categories of anomalies and the risk of diarrheal disease. Exposure to extreme cold was associated with increases in diarrheal disease risk in all age groups by 11% (IRR, 1.11; 95% CI, 1.03 to 1.19) and in the under-5 group by 15% (IRR, 1.15; 95% CI, 1.07 to 1.24). Likewise, extreme cold was associated with an increased viral diarrhea rate in the under-5 age group (IRR, 1.31; 95% CI, 1.01 to 1.70). Exposure to cold elevated the risk of bacterial diarrhea in all age groups by 9% (IRR, 1.09; 95% CI, 1.01 to 1.18) and in the under-5 group by 6% (IRR, 1.06; 95% CI, 1.01 to 1.13). Likewise, heat and extreme heat were associated with elevated rates of all infectious diarrhea, but the results were no longer significant when the analysis was further broken down into bacterial and viral diarrhea. Exposure to hotter conditions was associated with increases in diarrheal risk among all age groups (IRR, 1.03; 95% CI, 1.02 to 1.13) and in the under-5 group (IRR, 1.03; 95% CI, 1.02 to 1.12). Extreme heat increased the risk of all-cause infectious diarrhea in the under-5 age group (IRR, 1.18; 95% CI, 1.16 to 1.40) and in all ages (IRR, 1.08; 95% CI, 1.04 to 1.25).

This study did not find any significant positive association between extreme anomaly precipitation events and all-cause infectious diarrhea (Table 3). However, we observed a significant protective effect of wet conditions on viral diarrhea in the under-5 age group by 18% (IRR, 0.82; 95% CI, 0.70 to 0.95). Likewise, we did not observe a positive association of extreme anomaly SWS events on all-cause infectious diarrhea in this study. We found that extremely dry conditions were associated with all infectious and viral diarrhea among all study groups, with the highest reduction of 47% (IRR, 0.53; 95% CI, 0.39 to 0.73) on viral diarrhea in all age groups. We also observed a significant protective effect of drier conditions on viral diarrhea among all groups.

We also observed a strong positive association between viral diarrhea and Lunar New Year events, with risk ranging from 38% for all age groups (IRR, 1.38; 95% CI, 1.11 to 1.70) to 33% for the under-5 age group (IRR, 1.33; 95% CI, 1.07 to 1.65). In the seasonal analysis, except for a significant increase in the risk of bacterial diarrhea in the under-5 age group during the typhoon season (IRR, 1.12; 95% CI, 1.03 to 1.22), the risk of all studied diseases gradually decreased from winter to the typhoon season. Viral diarrhea showed the highest risk of cases during the winter season for both the all-age and under-5 age groups (IRR, 2.06; 95% CI, 1.70 to 2.51; and IRR, 1.61; 95% CI, 1.32 to 1.95, respectively).

## DISCUSSION

This is the first population-based study to evaluate the associations between all infectious and cause-specific diarrhea and climatic factors, including anomalies in temperature, precipitation, and SWS along with seasons. Our study identified an association between a +1°C anomaly in temperature and all infectious diarrhea, although some of the associations were no longer significant when the analysis was stratified by viral and bacterial diarrhea. Furthermore, viral diarrhea was significantly associated with a +1 mm SWS anomaly. As we defined the weather variables by percentile, both extreme cold and extreme heat increased the risk of all-cause infectious diarrhea in Taiwan. However, extremely dry and drier conditions were associated with a decreased risk of viral diarrhea. Interestingly, our study also identified the culturally important Lunar New Year event, which happens during the winter in Taiwan, as an important risk factor for infectious diarrhea.

This study identified a positive association between average temperature anomalies and all-cause infectious diarrhea in Taiwan at a lag of 2 months (Table 2). In addition, analyses based on threshold showed that hot and extremely hot conditions affected the risk of all infectious diarrhea. These findings are similar to those of a recent study that showed a 2.68% increase in outpatient visits for diarrhea in Shanghai for a 1°C increase in temperature [33]. Others have reported that higher temperatures can increase the risk of infectious diarrhea, potentially due to increases in the consumption of uncooked meat or spoiled food and can hasten bacterial growth [9,33,34]. A Korean study reported higher temperatures to be associated with salmonellosis and campylobacteriosis [34]. We also observed that anomalous extremely cold conditions, when the temperature was 2.59°C or more below the average temperature, affected the risk of all-cause infectious diarrhea and viral diarrhea. These findings are consistent with a recent study that reported associations between cold temperature and diarrhea in Taiwan, Hong Kong, and Japan [35]. A meta-analysis also reported a higher risk of viral diarrhea in colder temperature rather than hot temperatures [36]. Thus, an appropriate approach to address the risk of infectious diarrhea when the temperature is colder or warmer than the average values should be proposed accordingly.

We found there were no apparent effects of precipitation anomalies on the risk of infectious diarrhea in Taiwan. Our results are in contrast with a study conducted in Bangladesh that found a positive association between precipitation and reported typhoid cases at lags of 0-3 weeks, with 45% of total cases recorded during the monsoon period [37]. Prior studies have claimed that higher precipitation is associated with an increased risk of pathogens transmission through the drinking water system [38]. Increased turbidity and pathogen loads in the surface water are inevitable during the rainy season with higher precipitation due to overland runoff [39]. Conversely, our results showed a protective effect of higher precipitation anomalies for viral diarrhea. Previous studies have shown that the peak rainfall in Taiwan is observed from summer until fall, caused by the southwesterly monsoon flow that often bring typhoons along with the heavy rainfall [40,41]. Thus, the high precipitation during summertime might not be a favorable environment for viral diarrhea transmission in Taiwan. In addition, increased precipitation could lead to pathogen dilution and decrease the risk [42]. Further analysis should be carried out in the future to untangle the nebulous association between precipitation increments and diarrhea risk in different regions.

This study utilized SWS to represent all surface water, including both water constrained in water bodies and water overflowing onto the surrounding plains [21]. GFMS provides these data as the estimation of surface water depth (mm) above the land, which reflects recent flooding [20], and thus the risk of diarrhea. Our results indicated that a 1-unit increase in SWS elevated the risk of viral diarrhea. A recent study from Bangladesh showed higher frequencies of both cholera and non-cholera diarrhea r during flood periods [43]. Interestingly, this study found a protective effect of SWS when we broke down the analysis into categorical variables of SWS anomalies. This might be related to the sanitation conditions in Taiwan. A study in Taiwan revealed that local inhabitants have good water management, water literacy awareness, and behavior, as well as proper knowledge regarding drinking water safety and hygiene [44].

A prior study from Taiwan reported that the incidence of rotavirus infections had an epidemic peak in cooler months between January and March, supporting our findings [45]. The Taiwan Centers for Disease Control reported that viral gastroenteritis outbreaks were recorded in the emergency department during the Lunar New Year holiday in 2015, with the majority of cases being caused by rotavirus and norovirus (previously known as Norwalk virus) [46]. Due to family or friends gathering during the Lunar New Year event, the chances of transmission may increase [47], which corroborates our findings of the highest incidence of viral diarrhea during the Lunar New Year in Taiwan. The increase in the risk of infectious diarrhea during the winter season and Lunar New Year might also result from a decrease in temperature, which can enhance the replication and survival of diarrheal viruses [33]. Moreover, there are social and behavioral aspects of vulnerability to cold temperatures, as colder temperatures may alter the hygiene behavior among the population, leading to higher transmission of pathogens [48].

There are several strengths of this study, including its long temporal coverage (2004-2015) and comprehensive cause-specific outcome measure. This is the first study to link SWS with an increased diarrhea burden. This study successfully investigated associations between SWS/weather anomalies and cause-specific infectious diarrhea, which is more relevant in the context of climate change, using a negative binomial regression GEE model. Future studies incorporating other statistical modeling techniques, including a distributed lag nonlinear model, will furnish enriched epidemiological evidence linking cause-specific diarrheal disease and these novel climate change exposure metrics. Some limitations are noted as well. First, we did not control for confounding factors, such as socio-demographic details, as this individual-level information was not available. Second, we used a monthly temporal scale. Furthermore, the majority of the outpatient infectious diarrheal disease cases belonged to all-cause infectious diarrhea because clinical laboratory tests of the causative pathogen were not performed in outpatient departments most of the time, as stool testing for pathogens is done only for patients suffering from severe or moderate diarrhea. Despite these limitations, this study provides an in-depth assessment of vulnerability, which varied by age and the cause of diarrhea, including all infectious diarrhea, bacterial diarrhea, and viral diarrhea. The findings of the study can help in the development of appropriate mitigation strategies for infectious disease consequences under climate-change scenarios. Since extreme weather events are projected to increase despite mitigation efforts, we argue that data like ours should be used to develop location-specific early warning systems that can help communities adapt to the threats of climate change [49].

Our findings suggest that anomalies in SWS are significantly associated with the diarrhea burden in Taiwan, particularly for viral diarrhea. Moreover, extreme heat-related infectious diarrhea was most pronounced in the under-5 age group, while extremely cold months also elevated the risk of viral diarrhea in this age group. The winter season and Lunar New Year also increased the risk of all-cause infectious diarrhea and viral diarrhea, regardless of the age group. Since diarrheal disease continues to be a major cause of morbidity and mortality among young children, and climate change is leading to increases in extreme weather events, coordinated efforts are needed to enhance preparedness and management of diarrheal diseases.

## Figures and Tables

**Figure f4-epih-45-e2023024:**
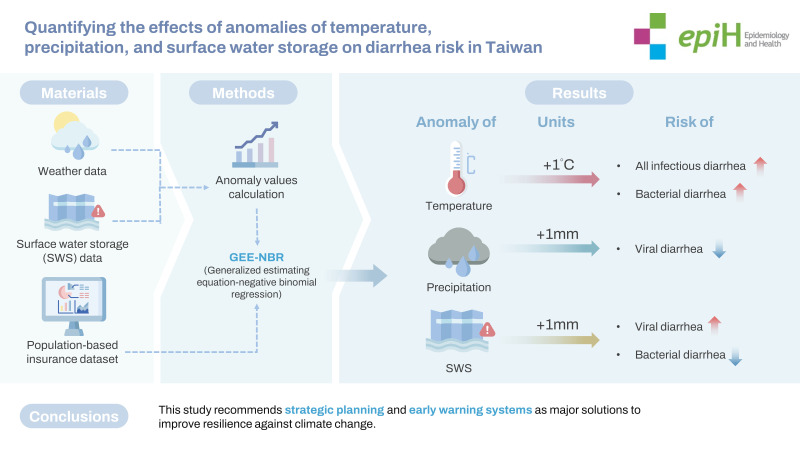


**Table 1.** Descriptive statistics of diarrhea cases for all ages and ages under 5, and observations and anomalies of weather variables in Taiwan from 2004 to 2015

Outpatient visits		Mean	Min	P5	P25	P50	P75	P95	Max
All ages									
	All infectious diarrhea	10,029	364	726	2,342	9,207	14,523	26,365	38,631
	Bacterial diarrhea	881	12	52	377	739	1,160	2,170	6,016
	Viral diarrhea	180	0	3	27	90	276	638	1,245
<5 yr									
	All infectious diarrhea	2,599	119	233	464	2,398	3,799	6,613	10,613
	Bacterial diarrhea	185	0	12	59	140	243	485	1,824
	Viral diarrhea	53	0	0	9	30	76	181	354
Weather anomaly factors									
	Average temperature (°C)	0.24	-2.60	-1.17	-0.25	0.23	0.73	1.69	4.08
	Precipitation (mm)	0.33	-11.81	-6.07	-2.08	-0.36	1.57	10.05	25.73
	Surface water storage (mm)	0.06	-2.27	-0.69	-0.19	-0.03	0.21	1.16	5.25
Weather factors									
	Average temperature (°C)	23.24	12.61	15.69	19.66	23.87	27.23	28.92	30.40
	Precipitation (mm)	178.24	0.00	9.30	46.32	110.74	248.58	582.28	1,187.63
	Surface water storage (mm)	1.11	0.16	0.34	0.52	0.84	1.40	2.69	4.25

P5, P25, P50, P75, and P95 refer to the 5th, 25th, 50th, 75th, and 95th percentiles, respectively. Min, minimum; Max, maximum.

**Table 2.** Age- and cause-specific incidence rate ratios (95% confidence intervals) of diarrhea associated with anomalies (adjusted for each other) in Taiwan from 2004 to 2015

Lag month			All infectious diarrhea		Bacterial diarrhea		Viral diarrhea	
			All ages	<5 yr	All ages	<5 yr	All ages	<5 yr
Annual event								
	Chinese New Year		1.18 (1.11, 1.24)^*^	1.13 (1.06, 1.20)^*^	1.12 (0.99, 1.21)	1.01 (0.92, 1.12)	1.38 (1.11, 1.70)^*^	1.33 (1.07, 1.65)^*^
Weather anomaly								
	Average temperature							
		Lag 0	0.97 (0.96, 0.99)	0.97 (0.95, 0.98)	1.00 (0.98, 1.03)	1.00 (0.97, 1.03)	1.03 (0.97, 1.10)	1.03 (0.96, 1.09)
		Lag 1	0.99 (0.97, 1.00)	1.00 (0.98, 1.02)	1.02 (0.99, 1.04)	1.04 (1.01, 1.07)^*^	1.04 (0.97, 1.11)	1.05 (0.99, 1.12)
		Lag 2	1.03 (1.01, 1.05)^*^	1.03 (1.01, 1.07)^*^	1.02 (0.99, 1.04)	1.04 (1.01, 1.07)^*^	1.02 (0.96, 1.08)	1.02 (0.96, 1.09)
	Precipitation							
		Lag 0	1.00 (1.00, 1.00)	1.00 (1.00, 1.00)	1.00 (1.00, 1.01)	1.00 (1.00, 1.01)	0.99 (0.98, 1.00)	0.99 (0.97, 1.00)
		Lag 1	1.00 (0.99, 1.00)	1.00 (0.99, 1.00)	1.00 (0.99, 1.00)	1.00 (1.00, 1.01)	0.98 (0.96, 0.99)^*^	0.97 (0.96, 0.99)^*^
		Lag 2	1.00 (1.00, 1.00)	1.00 (1.00, 1.00)	1.00 (0.99, 1.00)	1.00 (0.99, 1.01)	0.99 (0.97, 1.00)	0.98 (0.97, 1.00)
	Surface water storage							
		Lag 0	0.99 (0.97, 1.02)	0.98 (0.95, 1.01)	1.08 (0.99, 1.12)	0.97 (0.93, 1.02)	1.08 (0.97, 1.21)	1.12 (1.00, 1.25)
		Lag 1	0.98 (0.95, 1.01)	0.98 (0.95, 1.01)	1.04 (0.99, 1.09)	0.95 (0.90, 0.99)^*^	1.15 (1.03, 1.28)^*^	1.18 (1.06, 1.31)^*^
		Lag 2	1.00 (0.97, 1.02)	1.00 (0.97, 1.03)	1.08 (1.00, 1.09)	0.97 (0.92, 1.02)	1.22 (1.09, 1.36)^*^	1.27 (1.14, 1.42)^*^

* p<0.05.

**Table 3.** Age- and cause-specific incidence rate ratios (95% confidence intervals) of diarrhea associated with classifications of extreme weather conditions in Taiwan from 2004 to 2015

Variables		All infectious diarrhea		Bacterial diarrhea		Viral diarrhea	
		All ages	<5 yr	All ages	<5 yr	All ages	<5 yr
Temperature							
	Extremely cold	1.11 (1.03, 1.19)^*^	1.15 (1.07, 1.24)^*^	1.02 (0.92, 1.13)	1.02 (0.90, 1.15)	1.15 (0.88, 1.50)	1.31 (1.01, 1.70)^*^
	Cold	1.00 (0.97, 1.04)	1.04 (1.00, 1.08)	1.09 (1.01, 1.18)^*^	1.06 (1.01, 1.13)^*^	0.93 (0.81, 1.07)	0.97 (0.85, 1.11)
	Normal	1.00 (reference)	1.00 (reference)	1.00 (reference)	1.00 (reference)	1.00 (reference)	1.00 (reference)
	Hot	1.03 (1.02, 1.13)^*^	1.03 (1.02, 1.12)^*^	1.00 (0.95, 1.06)	1.04 (0.97, 1.10)	1.10 (0.95, 1.27)	1.16 (0.99, 1.34)
	Extremely hot	1.08 (1.04, 1.25)^*^	1.18 (1.16, 1.40)^*^	0.98 (0.87, 1.09)	0.98 (0.86, 1.13)	0.94 (0.70, 1.27)	1.11 (0.82, 1.49)
Precipitation							
	Extremely dry	1.02 (0.95, 1.10)	1.01 (0.94, 1.09)	1.09 (0.98, 1.21)	1.00 (0.88, 1.14)	1.03 (0.78, 1.36)	0.91 (0.69, 1.19)
	Dry	1.03 (0.99, 1.07)	0.99 (0.95, 1.03)	1.05 (0.99, 1.11)	1.01 (0.94, 1.08)	1.03 (0.89, 1.19)	0.95 (0.82, 1.10)
	Normal	1.00 (reference)	1.00 (reference)	1.00 (reference)	1.00 (reference)	1.00 (reference)	1.00 (reference)
	Wet	1.01 (0.97, 1.05)	0.97 (0.93, 1.01)	1.03 (0.97, 1.09)	0.97 (0.91, 1.04)	0.86 (0.74, 1.01)	0.82 (0.70, 0.95)
	Extremely wet	1.05 (0.97, 1.14)	1.04 (0.96, 1.13)	1.08 (0.96, 1.21)	1.08 (0.95, 1.24)	1.05 (0.78, 1.42)	0.84 (0.62, 1.13)
Surface water storage							
	Extremely dry	0.88 (0.81, 0.96)	0.87 (0.80, 0.94)	0.90 (0.80, 1.01)	0.91 (0.79, 1.04)	0.53 (0.39, 0.73)	0.57 (0.42, 0.77)
	Drier	0.99 (0.95, 1.03)	1.00 (0.96, 1.04)	1.01 (0.96, 1.07)	1.02 (0.95, 1.09)	0.85 (0.73, 0.98)	0.83 (0.71, 0.96)
	Normal	1.00 (reference)	1.00 (reference)	1.00 (reference)	1.00 (reference)	1.00 (reference)	1.00 (reference)
	Wetter	0.94 (0.90, 1.00)	0.92 (0.88, 1.00)	0.94 (0.88, 1.00)	0.93 (0.87, 1.00)	0.84 (0.72, 1.00)	0.92 (0.79, 1.07)
	Extremely wet	0.97 (0.89, 1.04)	0.92 (0.85, 1.00)	0.98 (0.87, 1.10)	0.93 (0.81, 1.06)	0.85 (0.63, 1.15)	0.87 (0.65, 1.17)
Season							
	Spring	1.21 (1.15, 1.28)^*^	1.10 (1.04, 1.16)^*^	1.12 (1.04, 1.21)^*^	0.88 (0.81, 0.97)	1.75 (1.43, 2.15)^*^	1.59 (1.30, 1.95)^*^
	*Mei-yu* (East Asian rainy season)	1.00 (reference)	1.00 (reference)	1.00 (reference)	1.00 (reference)	1.00 (reference)	1.00 (reference)
	Typhoon	1.02 (0.97, 1.07)	1.04 (0.99, 1.10)	1.07 (1.00, 1.15)	1.12 (1.03, 1.22)	0.92 (0.76, 1.11)	0.83 (0.69, 1.00)
	Autumn	1.10 (1.05, 1.15)^*^	1.14 (1.08, 1.19)^*^	1.09 (1.02, 1.17)^*^	1.08 (1.00, 1.17)^*^	1.20 (1.01, 1.43)^*^	1.06 (0.89, 1.26)
	Winter	1.36 (1.29, 1.43)^*^	1.21 (1.15, 1.27)^*^	1.18 (1.10, 1.27)^*^	0.89 (0.81, 0.97)	2.06 (1.70, 2.51)^*^	1.61 (1.32, 1.95)^*^

* p<0.05.

## SUPPLEMENTARY MATERIALS

Supplementary Material 1. Weather anomaly category by percentile

Supplementary Material 2. Quasi Information criterion (QIC) of various models configuration

Supplementary Material 3. Incident rate ratios (95% confidence interval) of univariate model of cause-specific diarrhea associated with anomaly-stratified by age in Taiwan, 2004-2016

Supplementary Material 4. Incident rate ratios (95% confidence interval) of univariate, multivariate, and model with interaction effects of cause-specific diarrhea associated with anomaly at lag 0 stratified by age in Taiwan, 2004-2016
