# Supplementary material for: Quantifying the effects of anomalies of temperature, precipitation, and surface water storage on diarrhea risk in Taiwan
Source: Epidemiol Health. 2023 Feb 15;45:e2023024. doi: 10.4178/epih.e2023024 (PMC10396799; doi:10.4178/epih.e2023024)
Supplement: Supplementary Material 1. — Weather anomaly category by percentile [file epih-45-e2023024-Supplementary-1.docx]

Supplementary Material 1. Weather anomaly category by percentile

| **Average temperature anomaly classification (°C)** | | |
| --- | --- | --- |
| Extreme cold | <5^th^ percentile | <-1.17 |
| Cold | **≥**5^th^ – <30^th^ percentile | -1.17–(-)0.14 |
| Normal* | **≥**30^th^– ≤70^th^ percentile | -0.13–0.61 |
| Hot | >70^th^ – ≤95^th^ percentile | 0.61–1.70 |
| Extreme hot | >95^th^ percentile value | >1.70 |
| **Precipitation anomaly classification (mm)** | | |
| Extreme dry | <5^th^ percentile | <-6.09 |
| Dry | **≥**5^th^ – <30^th^ percentile | -6.09–(-)1.57 |
| Normal * | **≥**30^th^– ≤70^th^ percentile | -1.56–0.80 |
| Wet | >70^th^ – ≤95^th^ percentile | 0.81-10.07 |
| Extreme wet | >95^th^ percentile value | >10.07 |
| **Surface water storage anomaly classification (mm)** | | |
| Extremely dry | <5^th^ percentile | <-0.69 |
| Drier | **≥**5^th^ – <30^th^ percentile | -0.69–(-)0.14 |
| Normal* | **≥**30^th^– ≤70^th^ percentile | -0.13–0.12 |
| Wetter | >70^th^ – ≤95^th^ percentile | 0.13–1.17 |
| Extremely wet | >95^th^ percentile value | >1.17 |
