# Supplementary material for: Quantifying the effects of anomalies of temperature, precipitation, and surface water storage on diarrhea risk in Taiwan
Source: Epidemiol Health. 2023 Feb 15;45:e2023024. doi: 10.4178/epih.e2023024 (PMC10396799; doi:10.4178/epih.e2023024)
Supplement: Supplementary Material 2. — Quasi Information criterion (QIC) of various models configuration [file epih-45-e2023024-Supplementary-2.docx]

Supplementary Material 2. Quasi Information criterion (QIC) of various models configuration

|  | All Infectious | | Bacterial | | Viral | |
| --- | --- | --- | --- | --- | --- | --- |
|  | Quasi information criterion (QIC) | | | | | |
| Model configuration | All age | U5 | All age | U5 | All age | U5 |
| Tavg | 4013.4 | 938.1 | 295.7 | 94.1 | 125.3 | 42.1 |
| Tavg+Precip | 3985.6 | 937.3 | 295.8 | 94.1 | 124.6 | 41.8 |
| Tavg+sws | 4171.7 | 936.2 | 296.9 | 93.8 | 125.7 | 41.9 |
| Tavg+sws+precip | 3978.5 | 937.0 | 295.5 | 93.6 | 124.5 | 41.3 |
| Tavg+Precip+Tavg*precip | 3989.2 | 933.6 | 295.6 | 94.1 | 125.3 | 41.9 |
| Tavg+sws+Tavg*sws | 4150.9 | 933.8 | 295.6 | 93.9 | 125.5 | 41.8 |
| Tavg+sws+precip+Tavg*precip+Tavg*sws | 3980.4 | 932.9 | 295.8 | 93.7 | 126.5 | 41.4 |
