# Supplementary material for: Quantifying the effects of anomalies of temperature, precipitation, and surface water storage on diarrhea risk in Taiwan
Source: Epidemiol Health. 2023 Feb 15;45:e2023024. doi: 10.4178/epih.e2023024 (PMC10396799; doi:10.4178/epih.e2023024)
Supplement: Supplementary Material 3. — Incident rate ratios (95% confidence interval) of univariate model of cause-specific diarrhea associated with anomaly-stratified by age in Taiwan, 2004-2016 [file epih-45-e2023024-Supplementary-3.docx]

Supplementary Material 3. Incident rate ratios (95% confidence interval) of univariate model of cause-specific diarrhea associated with anomaly-stratified by age in Taiwan, 2004-2016

| Weather Variables | Lag month | All infectious diarrhea | | | Bacterial diarrhea | | | Viral diarrhea | | |
| --- | --- | --- | --- | --- | --- | --- | --- | --- | --- | --- |
|  |  | All age | Under 5 years | All age | | Under 5 years | All age | | Under 5 years |  |
| Average temperature anomaly | Lag 0 | 1.01 (0.99, 1.03) | 1.00 (0.98, 1.03) | 0.95 (0.92, 0.98) | | 0.99 (0.96, 1.02) | 1.06 (1.00, 1.13) | | 1.02 (0.96, 1.08) |  |
|  | Lag 1 | 1.02 (1.00, 1.05) | 1.03 (1.01, 1.05) | 0.98 (0.95, 1.02) | | 1.03 (1.00, 1.06) | 1.07 (1.00, 1.13) | | 1.05 (0.99, 1.12) |  |
|  | Lag 2 | 1.04 (1.02, 1.06) | 1.04 (1.02, 1.07) | 0.99 (0.95, 1.02) | | 1.03 (1.00, 1.06) | 1.04 (0.98, 1.11) | | 1.01 (0.95, 1.07) |  |
| Precipitation anomaly | Lag 0 | 1.01 (1.00, 1.01) | 1.01 (1.00, 1.01) | 1.00 (1.00, 1.01) | | 1.00 (1.00, 1.01) | 0.99 (0.98, 1.00) | | 0.99 (0.98, 1.00) |  |
|  | Lag 1 | 1.00 (1.00, 1.00) | 1.00 (0.99, 1.00) | 1.00 (0.99, 1.00) | | 1.00 (0.99, 1.00) | 0.98 (0.97, 0.99) | | 0.98 (0.97, 0.99) |  |
|  | Lag 2 | 1.00 (1.00, 1.01) | 1.00 (1.00, 1.01) | 1.00 (0.99, 1.00) | | 1.00 (0.99, 1.00) | 1.00 (0.99, 1.01) | | 1.00 (0.99, 1.01) |  |
| Surface Water Storage (SWS) | Lag 0 | 1.04 (1.01, 1.08) | 1.03 (1.00, 1.07) | 1.06 (1.02, 1.12) | | 1.00 (0.96, 1.04) | 0.97 (0.89, 1.05) | | 1.05 (0.96, 1.15) |  |
|  | Lag 1 | 0.99 (0.96, 1.02) | 0.98 (0.95, 1.01) | 1.02 (0.97, 1.07) | | 0.99 (0.94, 1.05) | 0.96 (0.88, 1.05) | | 1.04 (0.95, 1.13) |  |
|  | Lag 2 | 1.01 (0.98, 1.05) | 1.01 (0.98, 1.04) | 1.05 (1.00, 1.10) | | 1.02 (0.97, 1.08) | 1.05 (0.96, 1.14) | | 1.18 (1.08, 1.29) |  |
