# Supplementary material for: Quantifying the effects of anomalies of temperature, precipitation, and surface water storage on diarrhea risk in Taiwan
Source: Epidemiol Health. 2023 Feb 15;45:e2023024. doi: 10.4178/epih.e2023024 (PMC10396799; doi:10.4178/epih.e2023024)
Supplement: Supplementary Material 4. — Incident rate ratios (95% confidence interval) of univariate, multivariate, and model with interaction effects of cause-specific diarrhea associated with anomaly at lag 0 stratified by age in Taiwan, 2004-2016 [file epih-45-e2023024-Supplementary-4.docx]

Supplementary Material 4. Incident rate ratios (95% confidence interval) of univariate, multivariate, and model with interaction effects of cause-specific diarrhea associated with anomaly at lag 0 stratified by age in Taiwan, 2004-2016

|  | All Infectious | | Bacterial | | Viral | |
| --- | --- | --- | --- | --- | --- | --- |
|  | All age | U5 | All age | U5 | All age | U5 |
| Tavg | | | | | | |
| Tavg | 1.00 (0.98, 1.02) | 1.00 (0.98, 1.02) | 0.99 (0.97, 1.02) | 0.99 (0.96, 1.02) | 1.04 (0.98, 1.10) | 1.02 (0.96, 1.08) |
| Tavg+Precip | | | | | | |
| Tavg | 1.01 (0.99, 1.03) | 1.01 (0.98, 1.03) | 0.99 (0.97, 1.02) | 0.99 (0.96, 1.02) | 1.03 (0.97, 1.09) | 1.01 (0.96, 1.08) |
| Precip | 1.01 (1.00, 1.01) | 1.01 (1.00, 1.01) | 1.00 (1.00, 1.01) | 1.00 (1.00, 1.01) | 1.00 (0.99, 1.01) | 0.99 (0.98, 1.00) |
| Tavg+sws | | | | | | |
| Tavg | 0.99 (0.97, 1.02) | 1.00 (0.98, 1.03) | 0.98 (0.96, 1.01) | 0.99 (0.96, 1.02) | 1.04 (0.98, 1.10) | 1.02 (0.96, 1.08) |
| sws | **1.05 (1.01, 1.08)** | **1.04 (1.00, 1.07)** | 1.00 (0.97, 1.04) | 0.98 (0.95, 1.02) | 1.04 (0.96, 1.13) | 1.01 (0.92, 1.09) |
| Tavg+sws+precip | | | | | | |
| Tavg | 0.97 (0.96, 0.99) | 0.97 (0.95, 0.98) | 1.00 (0.98, 1.03) | 1.00 (0.97, 1.03) | 1.03 (0.97, 1.10) | 1.03 (0.96, 1.09) |
| sws | 0.99 (0.97, 1.02) | 0.98 (0.95, 1.01) | 1.08 (0.99, 1.12) | 0.97 (0.93, 1.02) | 1.08 (0.97, 1.21) | **1.12 (1.01, 1.25)** |
| Precip | 1.00 (1.00, 1.00) | 1.00 (1.00, 1.00) | 1.00 (1.00, 1.01) | 1.00 (1.00, 1.01) | 0.99 (0.98, 1.00) | 0.99 (0.97, 1.00) |
| Tavg+Precip+Tavg*precip | | | | | | |
| Tavg | 1.01 (0.99, 1.03) | 1.01 (0.99, 1.03) | 0.99 (0.97, 1.02) | 0.99 (0.96, 1.02) | 1.03 (0.98, 1.09) | 1.02 (0.96, 1.08) |
| Precip | **1.01 (1.00, 1.01)** | 1.00 (1.00, 1.01) | 1.00 (1.00, 1.01) | 1.00 (1.00, 1.01) | 0.99 (0.98, 1.01) | 0.99 (0.98, 1.00) |
| Tavg * Precip | 1.01 (1.00, 1.01) | 1.01 (1.00, 1.01) | 1.00 (0.99, 1.01) | 1.00 (0.99, 1.00) | 1.01 (0.99, 1.02) | 1.00 (0.99, 1.02) |
| Tavg+sws+Tavg*sws | | | | | | |
| Tavg | 0.99 (0.97, 1.01) | 1.00 (0.98, 1.02) | 1.00 (0.97, 1.02) | 0.99 (0.96, 1.02) | 1.04 (0.98, 1.10) | 1.03 (0.97, 1.09) |
| sws | 1.03 (1.00, 1.07) | 1.03 (0.99, 1.06) | 1.00 (0.97, 1.04) | 0.99 (0.95, 1.03) | 1.05 (0.96, 1.14) | 1.04 (0.95, 1.14) |
| Tavg * sws | **1.08 (1.03, 1.14)** | 1.04 (0.99, 1.10) | 0.95 (0.90, 1.01) | 0.95 (0.89, 1.01) | 0.94 (0.82, 1.08) | 0.95 (0.83, 1.09) |
| Tavg+sws+precip+Tavg*precip+Tavg*sws | | | | | | |
| Tavg | 1.01 (0.99, 1.03) | 1.01 (0.99, 1.03) | 1.00 (0.97, 1.02) | 0.99 (0.97, 1.02) | 1.05 (0.99, 1.11) | 1.03 (0.97, 1.10) |
| Precip | 1.02 (0.98, 1.06) | 1.00 (1.00, 1.01) | 1.00 (1.00, 1.01) | 1.00 (1.00, 1.01) | 0.99 (0.97, 1.00) | 0.98 (0.97, 1.00) |
| sws | **1.01 (1.00, 1.01)** | 1.01 (0.97, 1.05) | 1.00 (0.95, 1.04) | 0.98 (0.93, 1.03) | **1.11 (1.00, 1.24)** | **1.14 (1.02, 1.27)** |
| Tavg * Precip | 1.01 (1.00, 1.01) | 1.01 (0.95, 1.07) | 1.00 (0.99, 1.01) | 1.00 (0.99, 1.01) | 1.01 (0.99, 1.03) | 1.01 (0.99, 1.03) |
| Tavg * sws | 1.00 (0.95, 1.06) | 1.01 (1.00, 1.02) | 0.94 (0.89, 1.01) | 0.95 (0.89, 1.02) | 0.89 (0.76, 1.04) | 0.91 (0.77, 1.07) |
